# Heterogeneity in gender dysphoria in a Brazilian sample awaiting gender-affirming surgery: a data-driven analysis

**DOI:** 10.1186/s12888-022-03727-3

**Published:** 2022-02-02

**Authors:** Dhiordan Cardoso Silva, Francisco Diego Rabelo-da-Ponte, Leonardo Romeira Salati, Maria Inês Rodrigues Lobato

**Affiliations:** 1 Transdisciplinary Gender Identity Program, Department of Psychiatry of Hospital de Clínicas de Porto Alegre, Porto Alegre, RS Brazil; 2 Graduate Program in Psychiatry and Behavioral Sciences, Medical School of the Federal University of Rio Grande do Sul, Rua Ramiro Barcelos, 2350, Porto Alegre, RS 90035-903 Brazil; 3 Molecular Psychiatry Laboratory, Department of Psychiatry of Hospital de Clínicas de Porto Alegre, Porto Alegre, RS Brazil

**Keywords:** Transgender Persons, Gender Dysphoria, Gender-affirming Surgery, Cluster Analysis, Unsupervised Machine learning

## Abstract

**Background:**

Population heterogeneity and the lack of clinical and sociodemographic information in transgender individuals with gender dysphoria (GD) remains a challenge for specialized services in mental health and surgical procedures. It aimed to identify and describe profiles in a sample waiting for gender-affirming surgery.

**Methods:**

A sample of 100 outpatients with GD was assessed through a structured interview, Emotion Regulation Difficulty Scale (DERS), Ruminative Response Scale (RRS), Depression, Anxiety and Stress Scale (DASS-21) and Life Satisfaction scale (SWLS). Cluster analysis was used to identify different profile categories.

**Results:**

Two subgroups with different profiles were identified: with less clinical severity (LCS) and with high clinical severity (HCS) on emotional dysregulation, acute symptoms of depression, anxiety, stress and association with mental rumination. The HCS cluster had greater vulnerability in terms of psychiatric history, use of psychotropic drugs, HIV positive, child abuse and suicidal behavior.

**Conclusion:**

Different profiles were found regarding the vulnerability to mental health in a sample of transgender people with GD who seek a public hospital service for the same clinical-surgical objective. Longitudinal studies are essential to monitor the impact of these contrasts and to target personalized therapeutic approaches in the prevention of psychiatric disorders.

## Background

The anguish and suffering experienced by many transgender individuals as a result of the incongruence between their assigned sex at birth and their current gender identity is clinically defined as gender dysphoria (GD) [1]. Worldwide, there is evidence of growth in the number of transgender people, but with broad differences in terms of prevalence. A meta-analysis has identified a prevalence rate for transsexualism of 4.6 in 100,000 individuals [2], while a more recent systematic review and meta-analysis has reported that transgender and gender-nonconforming people account for 1 to 30 individuals per 100,000 population. This underscores the discussion around the terminological heterogeneity as well as the characteristics of this population [3], especially if we take into account the lack of information about subgroups with GD and differences in the clinical presentation of these group.

In Brazil, there is a lack of information on transgender and gender-nonconforming people estimates and population profile, although a recent study has shown that gender diversity in the country covers about 2% of the adult population, almost three million people [4]. There are numerous limitations in tracing a GD epidemiological profile and estimating the need for specialized health services (especially involving surgical interventions) such as: terminological variability in describing transgender identities, different medical and surgical procedures are offered to individuals with GD globally [3]. Also, there is variation in the outcomes of interest reported by GD individuals and numerous sociodemographic (financial conditions, education and geographic location of residence, for example) and institutional factors may play as barriers in accessing health services specialized healthcare [1, 5, 6]. Costa et al. portrayed that institutional discrimination dynamics, lack of information and a design of political strategies that do not meet the specific health needs operate as barriers in accessing specialized services in the Brazilian context [7]. On the other hand, national studies endorse the importance of specialized care for this population, ranging from expanding care on risk factors for contracting sexually transmitted infections [8], minimizing impacts of traumatic events in childhood, studies on voice therapy, hormonal treatment [9], and impacts on quality of life after gender-affirming surgery [10].

Amidst this complex scenario, there is an urgent need to expand the qualification of methodologies for the assessment of psychological symptoms and the identification of profiles of individuals at greater emotional vulnerability so as to ensure the success of elective procedures. In addition to the available studies focusing on psychological factors in transgender and GD individuals [5, 11–16], there is a consensus regarding the importance of mapping and preventing psychiatric morbidities in the early stage of specialized treatments for gender-affirming surgery.

In medical research, machine learning has become a viable analytical resource that is increasingly used to identify patterns and diagnostic manifestations in multiple populations and clinical settings [17, 18]. The clustering may show multivariable traits and contributes to the understanding of clinical heterogeneity in specific groups under certain therapeutic conditions [19, 20]. To the best of our knowledge, this approach has not yet been used to group clinical, emotional, and sociodemographic characteristics associated with a GD diagnosis. The aim of this study is to identify subpopulations of people with GD in the context of care for surgical procedures of gender affirmation at a public university hospital in southern Brazil. In this way, check for patterns of characteristics that group participants and describe the subgroups. It is intended to contribute to specialized health care in GD, especially for those individuals who could benefit from targeted care management approaches.

## Method

We performed a cross-sectional study with outpatients diagnosed with GD, undergoing clinical follow-up before the gender-affirming surgery of a specialized public hospital service for transgender surgeries. Between December 2018 and January 2020, we recruited 100 individuals who were followed up at the specialized outpatient clinic in the city of Porto Alegre, who were awaiting the gender affirmation surgery and who met the following eligibility criteria: motivation to participate in the study, age of majority (18 years or more), established diagnosis of GD, cognitive conditions to respond to the interview, signature of the consent form and no use of psychoactive substances in the last week. Refusal to provide informed consent, inability to complete the scales or cognitive impairment, acute psychotic condition or use of illicit drugs in the last week were exclusion criteria from the study. The interviews were conducted by a psychiatrist and a psychologist specializing in the area of mental and trans health. Our study was approved by local ethical committee Hospital de Clínicas de Porto Alegre (Number: 73303717.2.0000.5327) and all participants provided informed consent.

### Instruments

Demographic data were collected through a semi-structured interview covering sociodemographic, clinical history and sexuality aspects. GD was established based on an evaluation by a psychiatrist trained in Diagnostic and Statistical Manual of Mental Disorders (DSM-5) [1] criteria and/or the corresponding I*nternational Classification of Diseases 11th* Revision (ICD-11) criteria for gender incongruence [21]. The following instruments were also administered:

Difficulties in Emotion Regulation Scale (DERS) [22, 23] (36-item version), divided into six subscales to assess difficulties in emotional regulation (or emotional dysregulation): impulse control difficulties, difficulty accepting emotional responses, lack of emotional awareness, difficulties engaging in goal-directed behavior when emotionally aroused, limited access to emotion regulation strategies, and lack of emotional clarity. Items are rated on a scale of 1 (“almost never”) to 5 (“almost always”). Higher scores indicate more difficulty in regulating emotions. The original version showed high internal consistency (α = 0.93) and good test-retest reliability [22].

Depression, Anxiety and Stress Scale - Short Form (DASS-21) [24, 25], a self-report measure, with 21 items that aim to assess the dimensions of the individual’s depressive, anxiety and stress symptoms referring to the last week (acute symptoms) through three subscales. The scale has an evaluative character in the interpretation of events and in the individual's physiological and psychological responses, with good internal consistency for each subscale (α = 0.92 for depression, α =0.90 for stress and α = 0.86 for anxiety) [20].

Ruminative Response Scale (RRS) [26, 27], used as a continuous variable to assess mental rumination, divided into brooding (involvement with symptoms of negative mood) and reflective (automatic focus; reflecting emotions maladaptive) dimension. The Cronbach's alpha coefficient for the weighting subscale was 0.72 and for brooding 0.77.

Satisfaction with Life Scale (SWLS) [28, 29] a 7-point scale, ranging from 1 (strongly disagree) to 7 (strongly agree) with questions that seek to globally measure the participants' judgment on satisfaction with their living conditions (Cronbach’s alpha α = 0.88).

### Data analysis

We performed cluster analysis in R (version 4.0.2), R Studio (version 3.5.3). After that, descriptive data analysis was performed using the IBM SPSS Statistics package, version 21. Cluster analysis was used to identify subgroups with homogeneous characteristics, using DERS and RRS results. We used the partition around medoids algorithm (PAM) (which builds a partition affecting observations to the nearest “medoids”, or the most representative individual in its cluster) to investigate the presence of subgroups in the GD sample using Gower’s distance through the R package ‘fpc’ (version 2.2.8). The same package was applied to compute the average silhouette width to determine the optimal numbers of clusters. The subgroups were compared regarding demographic, clinical, psychological, and behavioral measures. We checked the variables for outliers and normal distribution using the Kolmogorov–Smirnov test. The Mann-Whitney test and χ^2^ tests were performed as appropriate to compare the groups. Statistical significance was set at *p* < 0.05 (two-tailed) for all tests.

## Results

Sociodemographic, clinical, and gender identity a characteristic of subjects is presented in Table 1.

**Table 1:** Sociodemographic, gender identity and clinical characteristics (*n* = 100)

Characteristic	N	%
**Gender identity**		
Trans women	55	55
Trans men	45	45
**Ethnic self-declaration**		
Black	30	30.6
White	62	63.3
Native people	5	5.1
**History of maltreatment or neglect**	71	73.2
**History of Sexual violence**	25	25.8
**Hormone therapy**	83	83.6
**HIV positive**	17	17
**Psychiatric diagnoses past**	41	43.6
**Psychiatric diagnoses current**	16	17
**Psychiatric medication**	22	22.2
**History of drug dependence**	22	22.2
**Suicidal history**		
Suicidal ideation	75	75.8
Suicide plan	52	52.5
Suicide attempt	41	41.4
	**M [range]**	**SD**
**Age**	29.18 [18-53]	8.83
**Education (years)**	11.5 [9-14]	3.11
**Age who started HT**	21.59 [11-48]	7.09
**Cross-dressing – gender expression**	5.93 [3-12]	1.91
**Self-awareness of trans identity**	11.76 [5-30]	3.65
**Social transition of trans identity**	19.99 [11-48]	6.9

Descriptive data for sample characterization

The analysis of coefficients resulted in the retention of two groups (Table 2). The clusters were based on sociodemographic, clinical, and psychological data. Those with less symptomatology and more “adaptive” emotional skills identified with less clinical severity (LCS), and those with a higher proportion of symptomatic severity, with higher clinical severity (HCS). The “n valid” of tables vary according to the complete filling of the scales.

**Table 2:** Comparisons between DERS and DASS-21 clusters (*n* = 92)

	Cluster HCS (***n*** = 51)		Cluster LCS (***n*** = 41)			
Measure	Median	IQR	Median	IQR	U	***p***-value
**DERS scale**						
Goals	16	14 - 19	10	7-12	356.5	0.000
Nonacceptance	55	43.3 - 72.5	36.66	23.33 - 43.33	409	0.035
Impulse	15	13 - 21	9	7 - 10.5	252.5	0.000
Strategies	23	18.25 - 26.75	12	10 - 16	203.5	0.000
Awareness	17.5	13 - 21	15	11.5 - 18	794	0.000
Clarity	14.5	10.25 - 18.75	8	6 - 9.5	263.5	0.000
Total	22.52	20.31 - 27.68	15.05	12.66 - 17.77	101.5	0.000
**DASS – 21 scale**						
Anxiety score	4	2 - 8	1	0 - 2.5	532.5	0.000
Depression score	6	3-8.75	1	0 - 2	358	0.000
Stress score	7.5	5.25 - 12	4	2 - 7	527	0.000

*IQR* interquartile range, *U* Mann-Whitney test, *p* = < 0.05, *HCS* Cluster higher clinical severity, *LCS* Cluster less clinical severity, *DERS* Difficulties in Emotion Regulation Scale*, DASS-21* Depression, Anxiety and Stress Scale - Short Form

Through the DERS and DASS-21 scores, it is possible to differentiate the subgroups. With the exception of lack of emotional awareness (*p* = 0.035), emotional dysregulation was more gift in the HCS cluster (*p* = 0.000). The HCS cluster has a profile associated with higher scores for difficulties in emotional regulation strategies in general, that is - more difficulty in accepting emotional responses (non-acceptance), lack of emotional awareness (awareness), limited access to emotion regulation strategies (strategies), difficulties in engaging in goal-oriented behaviors (objectives), difficulties with impulse control (impulse), and lack of emotional clarity (clarity) [18]. Additionally, depression, anxiety, and stress scores were significantly lower in the LCS cluster (*p* = 0.000) for all variables analyzed, as shown in Table 2. The demographic, clinical, and emotional characteristics of the groups are shown in Tables 3 and 4 for categorical or continuous variables.

**Table 3:** Sociodemographic, gender identity and clinical aspects of the clusters (*n* = 92)

	Cluster HCS (***n*** = 51)		Cluster LCS (***n*** = 41)			
Continuous data	Median	IQR	Median	IQR	U	***p***-value
Age	25	21 - 31	30	25 - 37	832	0.012
Crossdressing (age)	6	5 - 7.75	5.5	4 - 7	948	0.267
Self-awareness (age)	12	9 - 15	12	9 - 14	1070	0.609
Social transition (age)	18	16 - 22.25	18	15 - 28	1110	0.710
Hormone therapy (age)	19	17 - 26	21	18 - 23	744	0.592
Education (years)	11	9 - 14	12	9 - 14	1138	0.751
Brooding domain (RRS)	13	12 - 16	9.5	7 - 12.25	459.5	0.000
Reflexion domain (RRS)	13	10 - 14.75	10	8 - 12	609.5	0.000
SWLS Scale	20.5	14 - 24	21	16.5 - 26.5	872.5	0.358

*IQR* interquartile range, *U* Mann-Whitney test, *p* < 0.05, *HCS* Cluster higher clinical severity, *LCS* Cluster less clinical severity, *RRS* Ruminative Response Scale subscales brooding and reflexion, *SWLS* Satisfaction with Life Scale

**Table 4:** Categorical aspects of the clusters (*n* = 100)

Categorized data	N	N Valid	HCS (%)	LCS (%)	X^**2**^	df	***p***-value
Gender identity	100						
Trans woman		55	49.1	50.9	1.257	1	0.262
Trans men		45	60.5	39.5			
Ethnic self-declaration	96						
White		61	61.1	66.7	2.052	3	0.562
Black		29	29.6	31			
Native		5	7.4	2.4			
Marital status	98						
Single - without stable relationship		67	70.9	65.1	0.374	1	0.541
With stable affective relationship		31	29.1	34.9			
Hormone therapy	97	81	77.8	90.7	2.901	1	0.089
HIV positive	97	15	7.4	25.8	6.048	1	0.014
History of substance dependence	97	21	20.4	23.3	0.117	1	0.732
History of psychiatric diagnosis	94						
Past		41	54.5	28.2	6.438	1	0.011
Current		16	23.5	9.8	3.001	1	
Psychiatric medication	97	22	31.5	11.6	5.381	1	0.020
Suicidal history	97						
Suicidal ideation		74	88.9	60.5	10.691	1	0.001
Suicide plan		51	64.8	37.2	7.316	1	0.007
Suicide attempt		41	51.9	30.2	4.585	1	0.032
Child sexual abuse self-report	97	37	44.4	30.2	2.049	1	0.152
History of maltreatment or neglect	95	70	83	61.9	5.387	1	0.020
Self-report of physical violence	95	24	22.6	28.6	0.436	1	0.509

*N valid* number of individuals analyzed, *HCS* Cluster higher clinical severity, *LCS* Cluster less clinical severity, *X*^*2*^ Chi-Square tests statistics, *df* bottom row, *p* < 0.05

The LCS cluster showed a difference of five years age in relation to the HCS cluster and significant association (*p*=0.012). Comparisons indicated that the HCS cluster has greater symptoms of depression, anxiety, stress and engagement with mental rumination (*p*=0.000 brooding and reflexion) (Table 3). In contrast to having a current mental health diagnosis, the past history of a psychiatric disorder was found to be associated between the clusters (*p* = 0.011), as well as the continued use of at least one psychiatric medication (*p* = 0.020). HIV positive was more frequent in the LCS cluster and revealed significance between the clusters (*p* = 0.014) (Table 4).

Overall satisfactions with life and hormone therapy (HT) were not statistically associated to clusters, even though the use of HT showed a trend. Self-report of childhood maltreatment were significantly more frequent in the HCS cluster and individuals of this cluster also showed greater involvement with suicidal ideation (*p*=0.001), planning suicide (*p*=0.007), and suicide attempt (*p*=0.032) (Table 4).

## Discussion

To the best of our knowledge, this is the first study using cluster analysis to identify clinical GD subgroups in patients awaiting gender-affirming surgery in a specialized hospital service. Our aim is to identify whether there is a clinical heterogeneity among GD subjects, and other clinical and social factors associated to them. Overall, we identified two subgroups that differed in relation to emotional regulation and acute symptoms of depression, anxiety, and stress. Emotion regulation has been described in several clinical scenarios and listed as an important psychological dimension in transdiagnostic conditions [30, 31].

The HCS cluster demonstrated a greater association with maladaptive symptoms both difficulties with emotional regulation strategies, presence of depressive symptoms, anxiety, stress and mental rumination. Intense emotional reactions and emotional deregulation have been described in a range of psychiatric disorders, including depression, psychosis symptoms, anxiety, and dysfunctional behaviors typical of aggression and self-mutilation [32–34] and were described in studies with minority populations [35].

The specialized treatment of GD in the hospital context (i.e., gender-affirming surgery) is characterized for the complexity of integrated physical and mental health care [5]. Some studies have shown positive psychological impacts and well-being after gender-affirming surgery [10, 36, 37]. Conversely, other studies have reported suicide episodes during all phases of the gender transition [38] and after the surgical procedure [39, 40]. It should be noted, however, that there are inconsistencies in information about the phases of GD/gender-affirming surgery treatment and lack of details on the existence of psychiatric morbidities linked to these samples.

Our results evidenced an emotionally vulnerable group of individuals with risky behaviors such as suicide. A large proportion of the sample reported childhood maltreatment, reaching 83% in the HCS cluster. In a cross-sectional study exclusively with trans women in the same GD/ gender-affirming surgery context, researchers reported a relationship between childhood maltreatment and psychiatric morbidities [41]. A several reports have described psychiatric disorders in trans populations, mainly anxiety, mood, and psychotic disorders, especially in samples of trans women [13, 14, 16, 42].

Mental rumination was also significantly associated with HCS. Clinically, rumination is recognized as playing a role in mood symptoms, especially in the mediation of sadness in depressed mood and anxiety [27, 43] and also for its association with difficulties in decision-making and behavioral avoidance processes. It is believed that precisely because they are involved in rumination, HCS individuals end up having a vulnerability to towards emotional damage and more pronounced clinical symptoms. This cognitive dimension has been examined in transsexual women before and after gender-affirming surgery, and might be an important marker of psychological improvement after the surgical procedure [44].

Contrary to most other variables, which were associated with the HSC cluster, the use of HT revealed a trend toward association with the LCS cluster, including participants with more adaptive emotional skills. HT is recognized for minimizing the suffering of GD [5]. In a review of the literature of mental health in GD, a decrease in psychiatric vulnerabilities is observed as transgender people refer affirmative medical treatment, with variations across transition stages, especially in those who attend specialized health services [14].

Considering the results of this study, there appear to be at least two transgender groups with GD awaiting gender-affirming surgery: a group with less severe or more adaptive psychological symptoms that may or may not vary during follow-up; and another group with a more vulnerable clinical pattern. The latter group has a history of psychiatric disorders and clinical characteristics that warrant personalized medical and therapeutic care along with the specific needs of GD and surgical preparation. Our study has limitations: due to the size of the sample, we do not make comparisons between genders. In addition, some information was collected through interviews and confirmed in medical records without the application of specific instruments for suicidal behavior and abuse, for example. Conversely, this is the first use of a combination of empirical scales to identify dimensions such as emotional dysregulation, mental rumination, and symptoms of depression, anxiety, and stress in a sample with GD awaiting gender-affirming surgery.

In conclusion, based on a sample awaiting gender-affirming surgery in a specialized public service, we describe the presence of GD subgroups with more vulnerable characteristics that may have implications for a successful outcome in health. It is recommended that future research expand the longitudinal assessment of the repertoire of clinical characteristics and psychological symptoms in subgroups with GD in the medical and surgical environment such as that of gender-affirming surgery, as well as possible differences and impacts on mental health between groups. By using a machine learning methodology to synthesize the data, expected hope to have contributed to the reflection about the heterogeneity of the clinical manifestation of the GD, especially in the context of gender-affirming surgery.

## Conclusions

In conclusion, we identified two clusters with different profiles in a sample of individuals with GD awaiting gender-affirming surgery. One of the clusters was associated more difficulties in emotional regulation, greater acute symptoms of depression, anxiety and stress, high mental rumination scores, higher vulnerability in terms of psychiatric history, use of psychotropic drugs, HIV positive, child abuse history and suicidal behavior. Longitudinal studies are needed to examine the impact of these pattern differences in the treatment of GD, gender-affirming surgery and to support personalized strategies for prevention/promotion of mental health. Although we consider advances in transgender health care in the Brazilian context, and we believe that worldwide, it is still recommended to evidence with data the health disparities in this population and subpopulations with greater vulnerability in mental health. Research of this nature can contribute to WPATH care guidelines and health promotion for this population.

## Data Availability

The datasets used in the current study are available from the corresponding author DCS on reasonable request.

## Abbreviations

DASS-21: Depression, Anxiety and Stress Scale - Short Form
DERS: Difficulties in Emotion Regulation Scale
DSM-5: Diagnostic and Statistical Manual of Mental Disorders
GD: Gender dysphoria
HCS: Higher clinical severity
HT: Hormone therapy
ICD-11: International Classification of Diseases 11th Revision
LCS: Less clinical severity
PAM: Partition around medoids
RRS: Ruminative Response Scale
SWLS: Satisfaction with Life Scale
WPATH: World Professional Association for Transgender Health
